# Oatp1 Enhances Bioluminescence by Acting as a Plasma Membrane Transporter for d-luciferin

**DOI:** 10.1007/s11307-014-0741-4

**Published:** 2014-05-06

**Authors:** P. Stephen Patrick, Scott K. Lyons, Tiago B. Rodrigues, Kevin M. Brindle

**Affiliations:** 1 Department of Biochemistry, University of Cambridge, Tennis Court Road, Cambridge, UK CB2 1QW; 2 Cancer Research UK Cambridge Institute, Li Ka Shing Centre, University of Cambridge, Robinson Way, Cambridge, UK CB2 0RE

**Keywords:** Bioluminescence, Molecular imaging, Luciferase, Reporter gene, Oatp1

## Abstract

**Purpose:**

Bioluminescence imaging is a powerful tool for studying gene expression and cell migration in intact living organisms. However, production of bioluminescence by cells transfected to express luciferase can be limited by the rate of plasma membrane transport of its substrate d-luciferin. We sought to identify a plasma membrane transporter for d-luciferin that could be expressed alongside luciferase to increase transmembrane flux of its substrate and thereby increase light output.

**Procedures:**

Luciferase-expressing cells were transfected with a lentivirus encoding the rat reno-hepatic organic anion transporter protein, Oatp1, which was identified as a potential transporter for d-luciferin. Light output was compared between cells expressing luciferase and those also expressing Oatp1.

**Results:**

In two cell lines and in mouse xenografts, co-expression of Oatp1 with luciferase increased light output by several fold, following addition of luciferin.

**Conclusions:**

The increase in light output thus obtained will allow more sensitive detection of luciferase-expressing cells *in vivo*.

**Electronic supplementary material:**

The online version of this article (doi:10.1007/s11307-014-0741-4) contains supplementary material, which is available to authorized users.

## Introduction

Bioluminescence imaging is a versatile and widely available method for imaging gene expression and cell migration [1]. However, detection of cells expressing firefly luciferase *in vivo* is limited by scattering and absorption of the emitted light by proteins such as hemoglobin [2]. While this is less of a problem at relatively superficial body locations, it can reduce both the sensitivity and resolution with which internal organs and cell masses, such as early stage tumors, can be imaged [3]. Various modifications have been made to the luciferase reporter system to address this problem, including the following: red-shifted luciferases and luciferins, which show less light scattering [4–9], codon optimization to increase luciferase expression [10], and mutagenesis to increase enzyme stability [11] and activity [12]. However, with some exceptions [6, 10, 12], the majority of these strategies have not markedly increased light output *in vivo*.

Numerous studies have indicated that luciferin transport across the plasma membrane limits enzyme activity and hence light output. In an early study, light output of luciferase-expressing mammalian cells was increased by reducing the number of membranes that luciferin had to cross to reach the enzyme. This was achieved by removing the peroxisomal targeting sequence found in the native enzyme, thus localizing it to the cytoplasm [13–15]. However, despite this modification, the rate of luciferin transport across the plasma membrane still limited light output, with between 7 and 70 times more light produced by lysed bacterial and mammalian cells [16, 17]. Light output can also be increased by the addition of cell permeabilizing agents such as DMSO, which increase luciferin uptake [15]. Increasing the lipid solubility and membrane permeability of luciferin has also met with some success. For example, bioluminescent output in intact cells, but not lysed cells, was increased following incubation with a lipid-soluble ester derivative of luciferin [18]. However, the aqueous solubility of this substrate was much less than that of unmodified luciferin, reducing the practicality of delivering it *in vivo*. Amino-luciferin showed increased cell permeability in comparison to d-luciferin [19], resulting in a peak photon emission that was 25 % higher *in vivo*. However, the Vmax of luciferase for this substrate is up to ten times lower than for d-luciferin. Recent work has demonstrated that the cyclic alkylaminoluciferin, CycLuc1, produces several-fold more light than an equivalent dose of d-luciferin, providing greater sensitivity of detection in the brain, where d-luciferin uptake is low [20]. However, the ability of this substrate to give greater sensitivity than d-luciferin in other tissues, when the luciferin is used in its normal higher concentration range, remains to be evaluated since it is not yet widely available.

Luciferin uptake varies between different cell types in both plants and animals [21–24], and this has previously been explained by the existence of luciferin exporters [25, 26], though it could also be explained by the existence of as yet unidentified luciferin importers. A novel strategy to increase luciferin uptake, and hence light output in luciferase-expressing cells, would be to co-express a luciferin transporter together with luciferase. Although the existence of luciferin importer proteins has been inferred [27], to the authors’ knowledge, no importers of luciferin have been definitively identified. Bio-distribution studies have shown that luciferin concentrations are high in the liver and kidneys following intravenous and intraperitoneal injections in mice [21, 23], implying the presence of a protein or proteins that mediate luciferin uptake. The family of organic anion transporting polypeptides (OATP), of which rat (*Rattus norvegicus*) Oatp1 (also known as Slc21a1, Slco1a1), was the first member to be identified [28, 29], has over 60 members, which are expressed predominantly in the liver and kidneys and are responsible for the removal of a broad range of organic molecules from the blood, including xenobiotics, hormones, and toxins [30–32]. Among Oatp1’s substrates and inhibitors are organic anions and cations, many of which are, like d-luciferin [33], cyclic, polycyclic, or heteropolycyclic carboxylic acids [30, 34, 35].

The observation that Oatp1 has a broad range of substrates, the correlation between the location of Oatp1 expression and high luciferin uptake, and the resemblance of d-luciferin to some known substrates of Oatp1 suggested to us that expression of this transporter might facilitate cellular luciferin entry and thus enhance bioluminescent output. We found recently, when investigating Oatp1 as a potential gene reporter for use with magnetic resonance imaging (MRI) and single-photon emission computed tomography (SPECT) [36], that Oatp1 expression increased bioluminescence from cells expressing luciferase *in vivo*. We show here that this can be explained by Oatp1-mediated uptake of d-luciferin, which significantly enhances the sensitivity of this widely used gene reporter system.

## Materials and Methods

### Vectors

LV-PGK-SO, comprising a sequence encoding mStrawberry [36], E2A (QCTNYALLKLAGDVESNPGP) [37], and Oatp1 (kindly provided by Dr Allan Wolkoff, [GenBank: NM_017111.1]), with a stop codon at the end of Oatp1, was cloned into the lentiviral transfer plasmid pBOBI (Inder M. Verma laboratory, Salk Institute, California) downstream of a PGK promoter [38]. The control vector (referred to as LV-PGK-ST) contained Timd2 [Genbank: NM_001161356.1] in place of the Oatp1 sequence. Replication defective vesicular stomatitis virus glycoprotein (VSV-G) pseudotyped lentiviral vectors were produced [39] and purified [40] according to published protocols, using the transfer plasmids and three packaging plasmids (Inder M. Verma laboratory, Salk Institute, CA). Inducible plasmids were produced using a doxycycline responsive promoter (TRE3G) and the mStrawberry-E2A-Oatp sequence described above (referred to as pTRE3G-SO). Plasmid maps of these vectors are shown in additional files.

### Cell Culture

Human embryonic kidney cells (HEK 293T) were acquired from Thermo Scientific (Loughborough, UK), Lewis Lung murine carcinoma (LL2) cells from the American Type Tissue Collection (ATCC, Teddington, UK), and HCT 116 cells from LGC Promochem. HEK 293T and LL2 cells were grown in DMEM (Invitrogen, UK), supplemented with 10 % fetal bovine serum (FBS), 2 mM l-glutamine and 0.1 mM MEM nonessential amino acids. HCT 116 cells were grown in McCoy's 5A media with Glutamax (Invitrogen), supplemented with 10 % FBS. Cells were mycoplasma tested upon receipt.

### Cell Transfection and Transduction

Stable luciferase-expressing HEK 293T cells used to validate Oatp1 function were generated by transduction with a FIV-based vector [41] modified to encode a Luc2-YFP fusion protein (LHLY) under a CAGGS promoter and clones selected using hygromycin. LL2 cells were transfected with a pBluescript plasmid encoding luciferase under a CAGGS promoter. Stable Oatp1 (and control) expressing subclones were generated from the above by lentiviral transduction with LV-PGK-SO (and LV-PGK-ST) and dilution cloning. Transgene expression post-transduction was confirmed by Western blot and by flow cytometry (FACSCalibur II, BD Biosciences, CA, USA) or fluorescence imaging (IVIS 200 series camera; PerkinElmer) or by microscopy. The doxycycline-inducible mStrawberry-E2A-Oatp1 and luciferase-E2A-Oatp1 plasmids used for bioluminescence experiments *in vitro* were constructed using a modified TRE3G vector (Clontech) containing a puromycin resistance cassette for selection of stable clones.

### Western Blots

Cells were extracted using M-Per reagent (Thermo Scientific) with Complete Mini Protease inhibitors (Roche), and protein concentration determined using the Bradford assay. Extracts were run on an SDS PAGE gel (NuPAGE, Invitrogen), transferred to a nitrocellulose membrane and luciferase-YFP detected using polyclonal goat anti-luciferase antibody (g475A, Promega), at 1 in 25,000 dilution with incubation for 1 h at room temperature. For α-tubulin staining a monoclonal mouse anti-α-tubulin (T1799, Sigma-Aldrich) was used at 1 in 1,000 dilution with overnight incubation at 4 °C. For mStrawberry staining, rabbit polyclonal anti-RFP (ab34771, AbCam) was used at 1 in 10,000 dilution with overnight incubation at 4 °C. Horseradish peroxidase conjugated anti-rabbit (111-035-003), anti-goat (705-035-003-JIR), and anti-mouse (115-035-006-JIR) antibodies (Jackson ImmunoResearch, Suffolk, UK) were used as the secondary antibodies at 1 in 10,000 dilution with an incubation time of 45 min at room temperature. All antibodies were diluted with 5 % *w*/*v* milk powder and 0.1 % Tween.

### Imaging

**Fig. 1:**
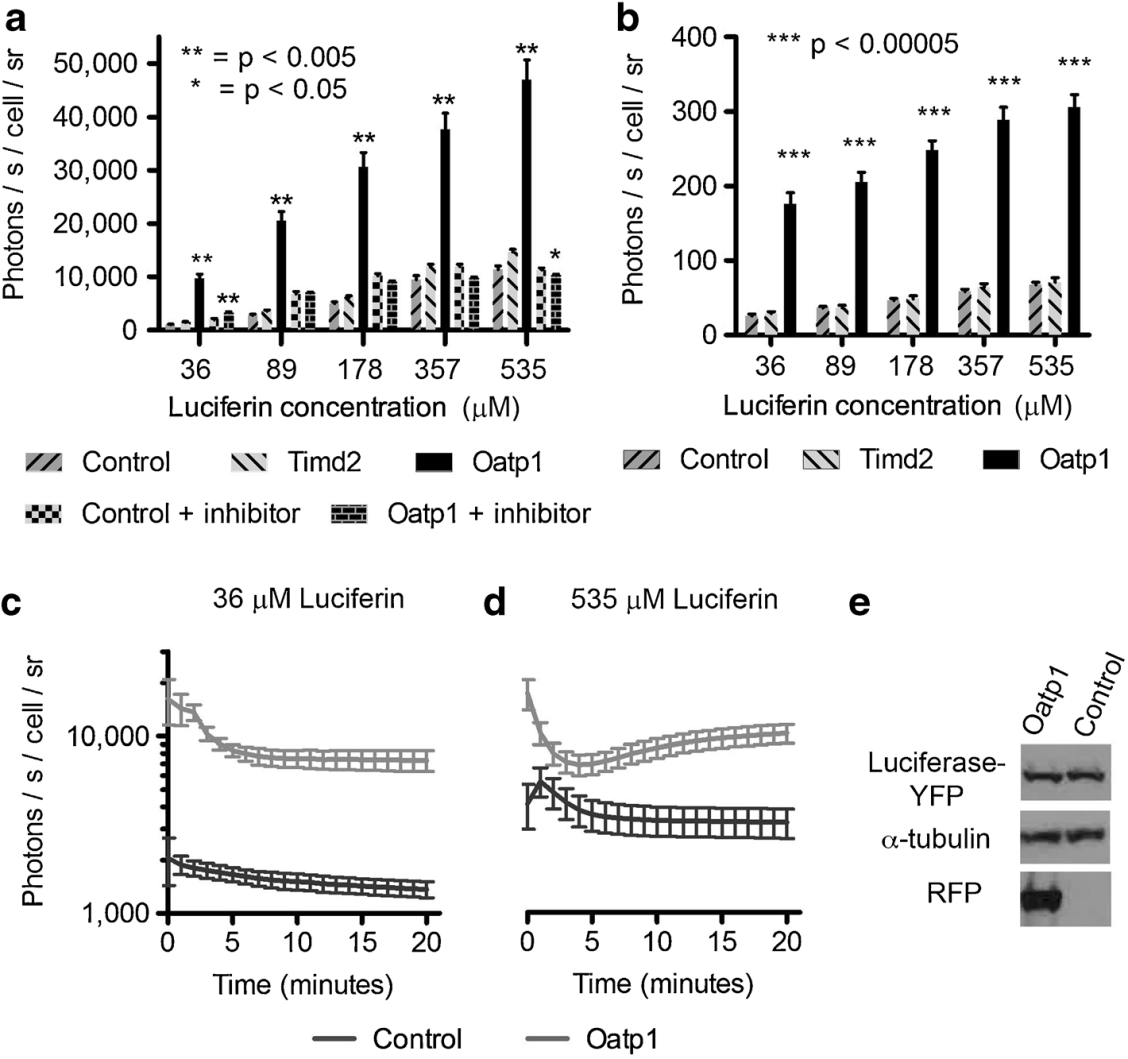
Oatp1 expression enhances bioluminescent output of luciferase-expressing cells. **a** HEK 293T cells expressing luciferase were transduced with a lentiviral vector expressing either Oatp1 or Timd2 (control) or were not transduced (control), 24 h before incubation with luciferin. Oatp1-expressing cells emitted significantly more light than control cells immediately after luciferin addition (1-s exposure time; *n* = 3, two-tailed *T* test). **b** Lewis lung carcinoma cells (LL2) expressing luciferase were transduced with lentiviral vectors, as described in (**a**). Clonal HEK 293T cells expressing Oatp1 produce more light than the luciferase-expressing cell population from which they were derived, for a period of 20 min following addition of luciferin at a concentration of **c** 36 μΜ and **d** 535 μM (*n* = 3, *error bars* show SD). **e** Western blot of protein extracts from the cells used in **c** and **d**, showing luciferase-YFP and RFP expression in control HEK 293T cells and HEK 293T cells transduced to express Oatp1. The presence of RFP indicates expression of the mStrawberry-Oatp1 transgene. *sr* steradian.

Cells were grown to 70 % confluence in six-well tissue culture plates (Nunc, Thermo Scientific) and then imaged using an IVIS 200 series camera (PerkinElmer) with small binning, a 1 s exposure, and an F-stop between 1 and 4, immediately after addition of luciferin to the growth medium (10 to 150 μg/ml; PerkinElmer). The final cell counts per well were typically between 2 and 3 million. For time course experiments (Figs. 1c, d, 3c, and 4a), measurements were repeated every minute for 20 min after luciferin addition. The number of cells per well was counted using a Z2 Cell and Particle Counter (Beckman-Coulter). The bioluminescence, expressed as photons/s/cell, was calculated for each well by dividing the photon count at each time point by the cell count for that well and an average calculated for four wells per condition. Inhibition experiments were performed by pre-mixing growth medium with quinine, at a concentration of 1 mM, and replacing the standard growth medium with this medium 5 min prior to addition of luciferin. Cell extracts were obtained by suspending 2 × 10^7^ cells in 1 ml of extraction buffer (100 mM potassium phosphate, pH 7.8, 1 mM dithiothreitol) [16], and freeze thawing three times on dry ice. The extract was mixed 2:1 with 3× assay buffer (25 mM glycylglycine buffer, pH 7.2, 5 mM ATP, and 15 mM MgSO4) [16] that had been premixed with luciferin. The number of photons per well was recorded at each time point using Living Image software (PerkinElmer). For imaging *in vivo*, xenograft-bearing mice were injected intraperitoneally with 150 mg/kg of luciferin and imaged every minute between 5 and 20 min post injection.

### Doxycycline Induction

Doxycycline was prepared as a stock solution of 10 mg/ml in water, sterile filtered, and stored at 4 °C. An appropriate volume of the stock solution was added to growth medium 24 h prior to imaging, to produce the final concentrations indicated in Figs. 3 and 4. Imaging of these cells was performed as described above.

### Statistical Analyses

Student’s *T* test and linear regression were performed using Excel (Microsoft) and Graphpad Prism (Graphpad Software Inc.) software. Unequal variance *T* tests were used when variance between groups were dissimilar. No blinding or randomization was used in any of the experiments, and no animals were excluded from analyses.

### Animals

Female SCID mice were obtained from Charles River (UK) at ~20 g (2 to 3 months) each and used within a few months of acquisition. Animals were injected subcutaneously with 1 × 10^7^ viable HEK 293T cells per flank and anesthetized using 2 % isoflurane in 75 % air/25 % O2 (flow rate 2 l/min) for bioluminescence and fluorescence imaging. Animal experiments were carried out under the authority of project and personal licenses issued by the Home Office, UK and were approved by the Cancer Research UK, Cambridge Institute Animal Welfare and Ethical Review Body.

## Results

### Oatp1 Expression Enhances Bioluminescence

A lentiviral vector (LV-PGK-SO) expressing the red fluorescent protein (RFP) mStrawberry and Oatp1 from the PGK promoter was constructed. The coding sequences of the two proteins were separated by an E2Asequence, which results in equimolar expression from the same transcript [42] (see Figure S1). Human embryonic kidney (HEK 293T) cells stably expressing a luciferase-YFP fusion protein [36], were transduced, at a multiplicity of infection of 2, with LV-PGK-SO, or with a control lentivirus, LV-PGK-ST, in which the coding sequence for Oatp1 was replaced with that of Timd2 [43]. Timd2 is a receptor that mediates H-ferritin endocytosis, and like Oatp1, is expressed on the plasma membrane in liver and kidney cells. Nontransduced cells were used as a further control. Expression of Oatp1, in cells transduced with LV-PGK-SO, and of Timd2, in cells transduced with LV-PGK-ST, was confirmed by observations of fluorescence from the co-expressed mStrawberry and by detection of mStrawberry on western blots. Cells expressing Oatp1 showed significantly higher initial rates of photon production per cell (three to sixfold), over a range of d-luciferin concentrations, when compared with cells expressing Timd2 or non-transduced cells (Fig. 1a), despite equivalent levels of luciferase expression. Comparison of cells expressing Timd2, non transduced cells and cells expressing Oatp1, using two-way ANOVA, showed that Oatp1 expression accounted for 57.8 % of the variation and d-luciferin concentration for 32.5 %, with 9.1 % interaction (*p* < 0.0001). In a comparison of the control groups, expression of Timd2 had no effect on light output (two-way ANOVA, *p* = 0.5526), with d-luciferin concentration accounting for 86 % of total variation (*p* < 0.0001) and Timd2 expression accounting for 0.04 %. The greatest increase in light output with Oatp1 expression, over sixfold, was observed at the lowest d-luciferin concentrations, the enhancement correlating negatively with d-luciferin concentration (Fig. 1a, b, c, d). Similar results were obtained with Lewis lung carcinoma (LL2) cells (Fig. 1b). There was a 4.5- to 6.2-fold increase in photon emission from LL2 cells expressing Oatp1, when compared to control cells. The enhancement again correlated negatively with d-luciferin concentration. Light output from Oatp1-expressing cells at 10 μg/ml of d-luciferin was 2.8× higher when compared to control cells incubated with 100 μg/ml (Fig. 1b). Quinine, an inhibitor of Oatp1 [35], decreased the light output of Oatp1-expressing HEK 293T cells to a level comparable with that of control cells incubated with the inhibitor (Fig. 1a).

Expression of Oatp1 led to sustained enhancement of bioluminescence following addition of 36 (10 μg/ml) (Fig. 1c) or 535 μΜ (150 μg/ml) luciferin (Fig. 1d) to luciferase-expressing HEK 293T cells that had been sub-cloned following transduction with the LV-PGK-SO vector. The usual burst kinetics associated with luciferase expression [16] were evident and were exaggerated by expression of Oatp1. Average light output from Oatp1-expressing cells was between 2.6- and 5.6-fold higher than control cells over the 20 min following luciferin addition. Expression of Oatp1 was confirmed by western blotting, which showed the presence of co-expressed mStrawberry (RFP). These blots also showed that luciferase-YFP was expressed equally in Oatp1-expressing and control cells (Fig. 1e).

### Effect of Oatp1 Expression on the Kinetics of Photon Emission

**Fig. 2:**
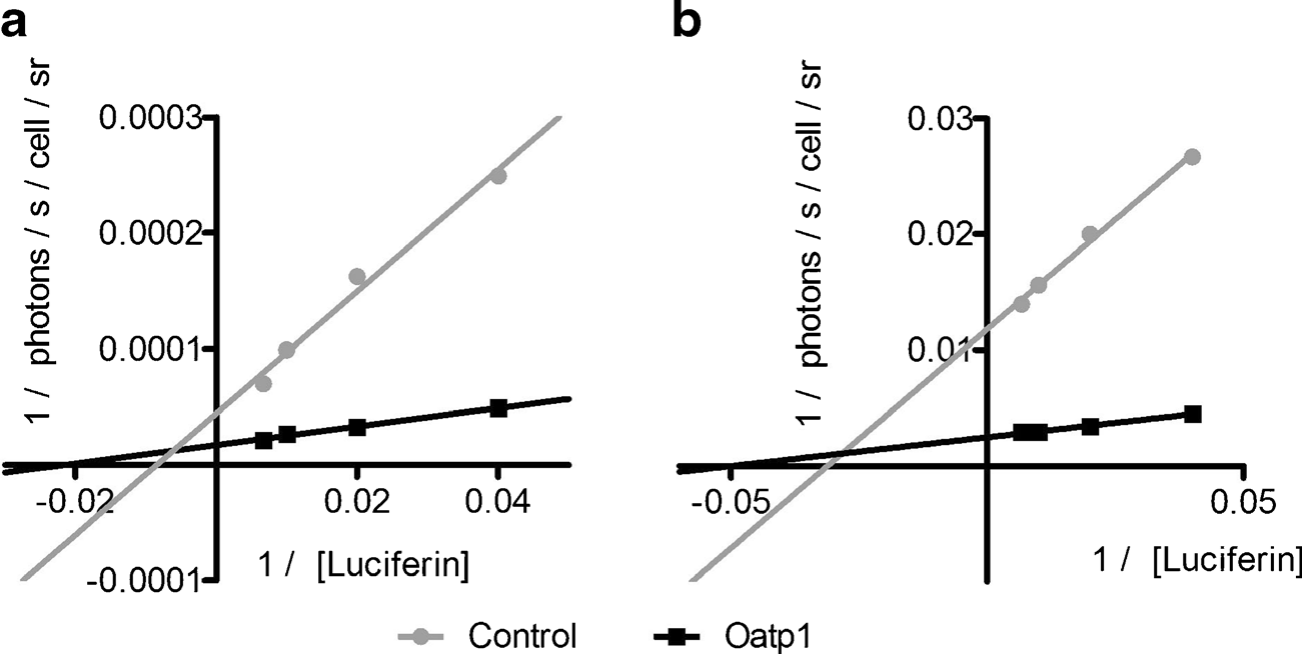
Effect of Oatp1 expression on the kinetics of photon production. Lineweaver-Burk plots showing the effects of Oatp1 expression and luciferin concentration on light output from **a** HEK 293T cells and **b** LL2 cells expressing luciferase-YFP. The data were taken from Fig. 1a, b.

Lineweaver-Burk plots of the initial rates of photon emission per cell (Fig. 2a, b) gave an apparent Km of luciferase for luciferin (Km (d-luciferin)) of 422 μM in control HEK 293T cells and 166 μM in cells expressing Oatp1. The Km in cell extracts was 57 μM. The apparent Km (d-luciferin) in LL2 cells was 114 μM in control cells and 71 μM in cells expressing Oatp1. Expression of Oatp1 increased Vmax in HEK 293T cells from 23,955 ± 5,791 to 58,770 ± 4,134 photons/s/cell (*p* < 0.05) and from 84 ± 4 photons/s/cell to 402 ± 17 photons/s/cell in LL2 cells (*p* < 0.01). These data confirm that cell uptake of luciferin is rate limiting for luciferase activity.

### Oatp1 Expression Correlates Linearly with Enhancement of Bioluminescence

**Fig. 3:**
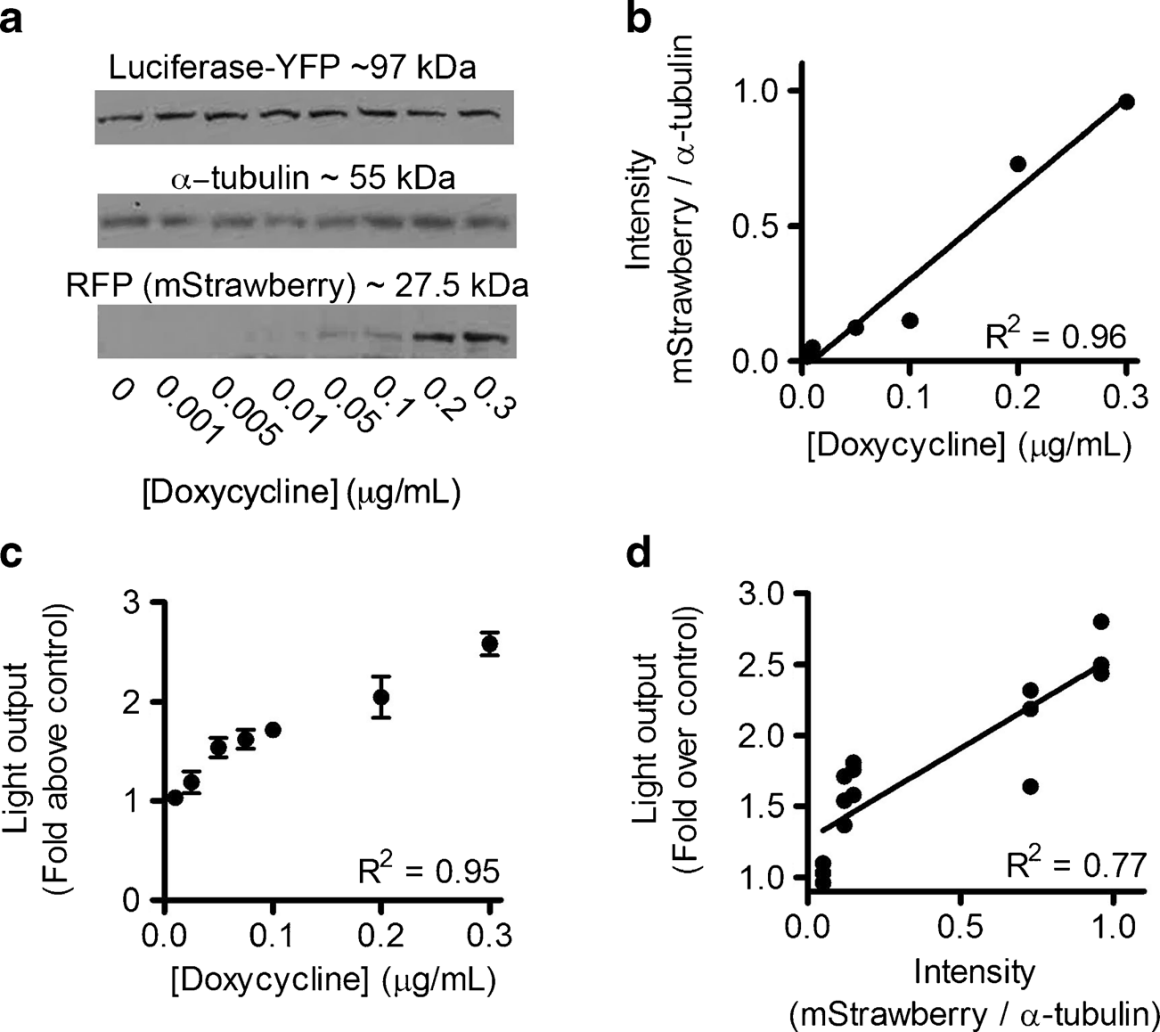
Oatp1 expression levels correlated linearly with enhancement of bioluminescence. HEK 293T cells expressing luciferase-YFP were transfected with a plasmid vector co-expressing mStrawberry and Oatp1 under the control of a doxycycline-inducible promoter (pTRE3G-SO). **a** Western blot showing induction of mStrawberry (RFP), and hence Oatp1, at increasing doxycycline concentrations. **b** The RFP band intensity on the western blot, when expressed as a ratio of the α-tubulin band intensity, showed a linear dependence on doxycycline concentration. **c** Enhancement of bioluminescence, following addition of luciferin (89 μM), was linearly dependent on the doxycycline concentration used to induce Oatp1 expression. **d** Enhancement of bioluminescence (**c**) *versus* Oatp1 expression (intensity of mStrawberry/α-tubulin on the Western blot (**b**)). All measurements were made 24 h after the addition of doxycycline to the cell culture medium.

The relationship between Oatp1 expression and enhancement of light output was further investigated in luciferase-YFP-expressing HEK 293T cells that had been stably transfected with a plasmid containing a doxycycline-inducible promoter driving expression of the mStrawberry-E2A-Oatp1 transgenes (pTRE3G-SO, see Supplementary Data File). There was a linear relationship between doxycycline concentration and an increase in Oatp1 expression, as determined from the levels of co-expressed mStrawberry on Western blots (Fig. 3a, b). Western blotting also showed that there was no change in the expression of luciferase-YFP with increasing doxycycline concentration (Fig. 3a). There was also a linear relationship between doxycycline concentration and enhancement of bioluminescence (Fig. 3c) and thus between bioluminescence enhancement and Oatp1 expression (Fig. 3d).

**Fig. 4:**
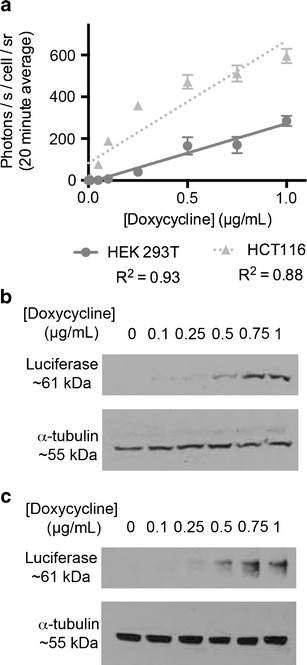
Co-induction of luciferase and Oatp1 expression correlates with enhancement of bioluminescence. HEK 293T and HCT116 cells were transfected with a plasmid (pTRE3G-LO) expressing luciferase and Oatp1 from a doxycycline-inducible promoter. **a** Enhancement of bioluminescence, following addition of luciferin (89 μΜ), was dependent on the doxycycline concentration used to induce expression (*n* = 3, *error bars* show SD). Western blot showing induction of luciferase, and hence Oatp1, at increasing doxycycline concentrations in **b** HEK 293T cells and **c** HCT116 cells.

Similar experiments were performed in which both luciferase and Oatp1 were co-expressed from a doxycycline-inducible promoter (Fig. 4). HEK 293T and HCT116 cells were stably transfected with pTRE3G-LO (see Supplementary Data File), in which the luciferase and Oatp1 coding sequences were separated by an E2A sequence [37]. In both cell lines photon emission was correlated with the concentration of doxycycline used to induce gene expression (Fig. 4a). Induction of both luciferase and Oatp1 was confirmed by measurements of increased luciferase expression on Western blots (Fig. 4b, c). Increased light output appeared to correlate linearly with Oatp1 expression in HEK 293T cells; however, there was some evidence of nonlinearity in HCT116 cells (Fig. 4a). This may be due to variation in expression of luciferin exporter proteins of the ABCG2 or BCRP families [24, 25], or constitutive OATP1B3 expression in HCT116 cells [44].

### Oatp1 Expression Increased Bioluminescence *In Vivo*

**Fig. 5:**
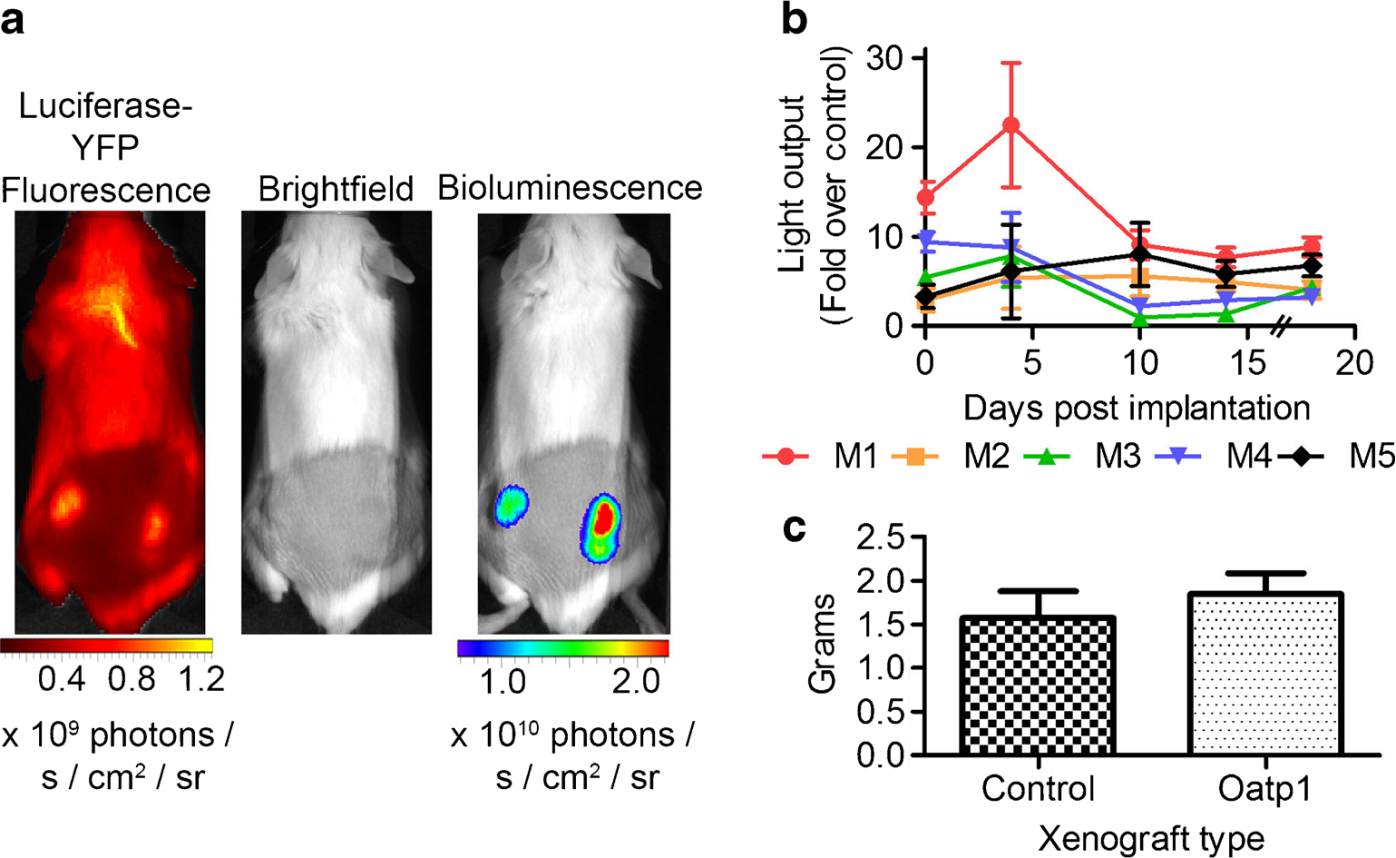
Oatp1 expression increases luciferase bioluminescence *in vivo*. **a** Representative mouse showing increased bioluminescence from the Oatp1-expressing HEK 293T xenograft (*right flank*), when compared with the control xenograft, expressing luciferase-YFP alone (*left flank*). YFP fluorescence demonstrated that luciferase was equally expressed in both xenografts. **b** Enhancement of light output of individual Oatp1-expressing xenografts relative to the contralateral control xenograft. *Points* show the ratio of the mean light output between 5 and 20 min following luciferin injection, for up to 18 days post cell implantation. *Error bars* show ± SD. At the 18 day time point, the light outputs of the Oatp1-expressing and control xenografts were normalized to the respective weights of the excised tissues at 24 days post cell implantation. Light output was significantly greater at all time points in Oatp1-expressing xenografts compared to control xenografts (*n* = 5, paired one-tailed *T* test), *p* < 0.05 for all time points. **c** Wet weight of excised control and Oatp1-expressing HEK 293 T xenografts 24 days after cell implantation.

We have demonstrated previously that Oatp1 expression increases light output *in vivo* [36]. Control HEK 293 T cells (1 × 10^7^) expressing luciferase-YFP (the same clone as used in Fig. 1) were implanted in the left flank of SCID mice, and 1 × 10^7^ of the same cells, which had transduced with a lentiviral vector expressing Oatp1 and mStrawberry and then cloned (shown in Fig. 1c, d, e), were implanted in the right flank (*n* = 5). Animals were imaged every minute between 5 and 20 min following intraperitoneal injection of luciferin (150 mg/kg). A representative bioluminescence image from a mouse at day 15 post implantation is shown in Fig. 5a. Corresponding fluorescence images showed that luciferase-YFP was expressed equally in the contralateral xenografts. Light output from xenografts expressing Oatp1 in five different animals, averaged over 15 min following luciferin injection, was between ~5 and up to 20-fold higher than the corresponding contralateral control xenografts for up to 18 days post implantation of the cells (see Fig. 5b) [36]. Given the negative correlation between bioluminescence enhancement and d-luciferin concentration observed in the cell experiments this wide variation in enhancement observed *in vivo* may reflect differences in the delivery of luciferin to the tissue. The previous studies, in which we investigated the use of Oatp1 as a gene reporter for MRI and for SPECT, demonstrated that Oatp1 expression, at the levels used here, had no measureable effect on cell growth *in vitro* [36]. Additionally, the ability of HEK 293T cells to form xenografts and their subsequent growth *in vivo* was not influenced by Oatp1-expression. There was no significant difference in the weights of excised control and Oatp1-expressing HEK 293T xenografts 24 days after cell implantation (*n* = 5, two-tailed paired *T* test, *p* = 0.13) (see Fig. 5c).

## Discussion

A major limitation of luciferase as a reporter gene has been identified as the rate of luciferin uptake into cells [15]. This has been addressed previously by lowering pH, so that luciferin becomes protonated and more membrane-soluble, or by adding DMSO to permeabilize the plasma membrane. However, these approaches damage cell viability [15] and cannot be used *in vivo*, in an intact animal. Another approach has been to develop synthetic luciferins with increased membrane permeability [20]. We have addressed this problem here by identifying a plasma membrane transporter for luciferin, which when expressed with luciferase enhances light output by several-fold. Our approach would also be compatible with other approaches that have been used to improve light output, such as codon optimization and red-shifting of the emitted spectrum [6, 10], as well as future gains in light output that might be achieved by directed evolution of the enzyme. However, it should be noted that the enhancement of light output will likely be influenced by a number of factors in addition to the levels of Oatp1 transgene expression, including luciferin concentration, the stage of xenograft development, and the endogenous levels of expression of luciferin importers, such as Oatp1 [29–32], and also exporters [25, 26].

The enhancement demonstrated here should facilitate bioluminescent detection of labeled cells in situations where bioluminescent output is low, for example, when there are few labeled cells or they are located at a greater tissue depth. For example, detection of the very earliest stages of tumorigenesis in mouse models of cancer following the implantation of small numbers of tumor stem cells or the early stages of autochthonous tumor development following the transformation of somatic cells using viral vectors [45]. Enhanced light output could also increase the sensitivity of detection of gene expression in regions where luciferin uptake is low, such as the brain [21, 23] and detection of luciferase expression when relatively weak promoters are used. The latter problem has been addressed previously by using a two-step transcriptional amplification (TSTA) system [46–48]. This system, together with the one described here, could further improve light output and the sensitivity of detection of weak promoter activation. Co-expression of Oatp1 could also be used to reduce the amount of luciferin required.

Bioluminescence measurements on tumor cells expressing luciferase *in vivo* have been used to assess response to new therapeutic drugs [49]. Since Oatp1 is known to transport a wide range of different molecules, its potential to transport the drug molecule under investigation would need to be considered when assessing the pharmacodynamic properties of the drug.

## Conclusions

We demonstrated recently that, by transporting a clinically approved hepatotropic contrast agent, Oatp1 can be used as a gene reporter for MRI and that by exchanging the Gd^3+^ ion in this contrast agent for ^111^In, can be used with SPECT as well [36]. Together with its capability to increase luciferase-dependent bioluminescence, this makes Oatp1 a very versatile system for imaging gene expression *in vivo*.

## Electronic supplementary material

Below is the link to the electronic supplementary material.

ESM 1 (PDF 1401 kb)
