# Supplementary material for: Oatp1 Enhances Bioluminescence by Acting as a Plasma Membrane Transporter for d-luciferin
Source: Mol Imaging Biol. 2014 May 6;16(5):626–34. doi: 10.1007/s11307-014-0741-4 (PMC4161938; doi:10.1007/s11307-014-0741-4)
Supplement: Supplementary file 1 — (PDF 1401 kb) [file 11307_2014_741_MOESM1_ESM.pdf]

Supplemental File

Oatp1 enhances bioluminescence by acting as a plasma membrane transporter for D-luciferin

Molecular Imaging and Biology

P. Stephen Patrick[1,2], Scott K. Lyons[2], Tiago Rodrigues[2], Kevin M. Brindle*[1,2]

[1]Department of Biochemistry, University of Cambridge, Tennis Court Road, Cambridge, UK, CB2 1QW

[2]Cancer Research UK Cambridge Institute, Li Ka Shing Centre, University of Cambridge, Robinson Way, Cambridge, CB2 0RE

*Corresponding author: Prof. Kevin M. Brindle, Department of Biochemistry, University of Cambridge, Tennis Court Road, Cambridge, UK, CB2 1QW

Phone +44 (0)1223 333674.

Email: kmb1001@cam.ac.uk

Figure S1

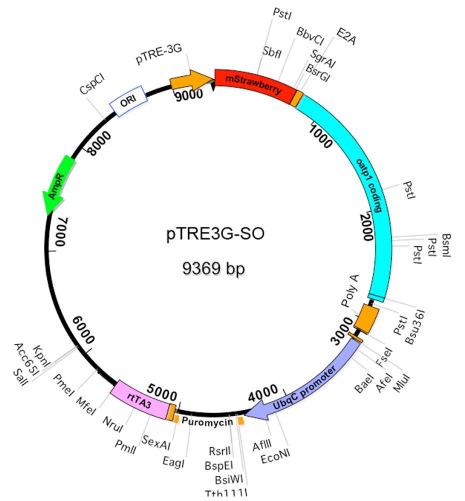

Name	Freq	Position
Acc65I	1	6977 (11566)
ApaI	1	3122 (11566)
BlpI	1	2820 (11566)
BsaBI	1	3066 (11566)
BsrGI	1	3837 (11566)
BstZ17I	1	9372 (11566)
Bsu36I	1	5889 (11566)
ClaI	1	2509 (11566)
CspCI	1	628 (11566)
CspCI'	1	663 (11566)
FseI	1	9012 (11566)
FspAI	1	3200 (11566)
HpaI	1	3126 (11566)
KpnI	1	6981 (11566)
MauBI	1	8772 (11566)
MluI	1	230 (11566)
NotI	1	1521 (11566)
PacI	1	2518 (11566)
PspOMI	1	3118 (11566)
SanDI	1	2307 (11566)
SbfI	1	3492 (11566)
SfiI	1	3117 (11566)
SnaBI	1	592 (11566)
XbaI	1	3058 (11566)
XcmI	1	3823 (11566)

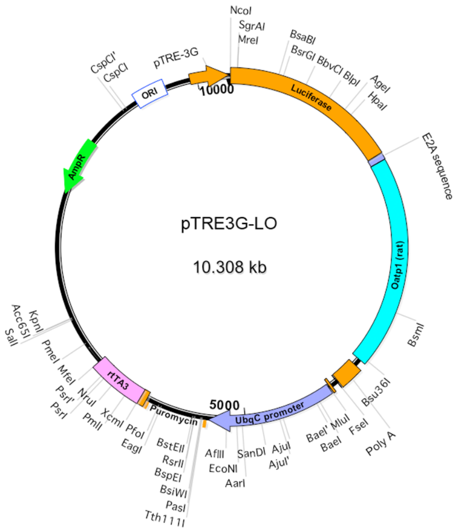

Name	Freq	Position
AarI	1	5034 (10308)
Acc65I	1	7037 (10308)
AflII	1	5180 (10308)
AgeI	1	1146 (10308)
AjuI	1	4652 (10308)
AjuI'	1	4684 (10308)
BaeI	1	4282 (10308)
BaeI'	1	4315 (10308)
BbvCI	1	726 (10308)
BlpI	1	969 (10308)
BsaBI	1	462 (10308)
BsiWI	1	5425 (10308)
BsmI	1	3190 (10308)
BspEI	1	5482 (10308)
BsrGI	1	505 (10308)
BstEII	1	5503 (10308)
Bsu36I	1	3706 (10308)
CspCI	1	9264 (10308)
CspCI'	1	9229 (10308)
EagI	1	5817 (10308)
EcoNI	1	5084 (10308)
FseI	1	4043 (10308)
HpaI	1	1220 (10308)
KpnI	1	7041 (10308)
MfeI	1	6549 (10308)
MluI	1	4136 (10308)
MreI	1	78 (10308)
NcoI	1	13 (10308)
NruI	1	6312 (10308)
PasI	1	5418 (10308)
PfoI	1	5844 (10308)
PmeI	1	6765 (10308)
PmlI	1	6163 (10308)
PsrI	1	6407 (10308)
PsrI'	1	6439 (10308)
RsrII	1	5485 (10308)
SalI	1	7023 (10308)
SanDI	1	4855 (10308)
SgrAI	1	78 (10308)
Tth111I	1	5411 (10308)
XcmI	1	6037 (10308)

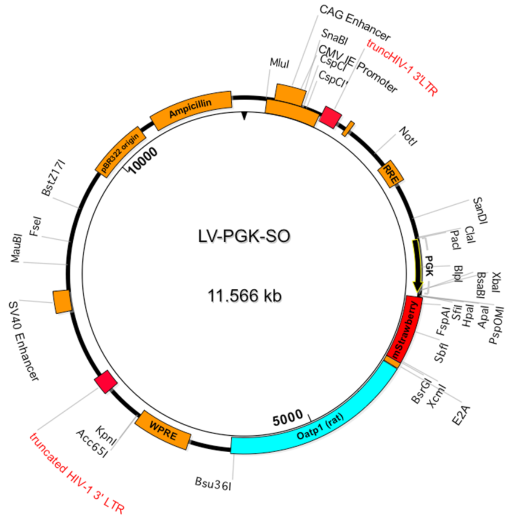

Name	Freq	Position
Acc65I	1	6977 (11566)
ApaI	1	3122 (11566)
BlpI	1	2820 (11566)
BsaBI	1	3066 (11566)
BsrGI	1	3837 (11566)
BstZ17I	1	9372 (11566)
Bsu36I	1	5889 (11566)
ClaI	1	2509 (11566)
CspCI	1	628 (11566)
CspCI'	1	663 (11566)
FseI	1	9012 (11566)
FspAI	1	3200 (11566)
HpaI	1	3126 (11566)
KpnI	1	6981 (11566)
MauBI	1	8772 (11566)
MluI	1	230 (11566)
NotI	1	1521 (11566)
PacI	1	2518 (11566)
PspOMI	1	3118 (11566)
SanDI	1	2307 (11566)
SbfI	1	3492 (11566)
SfiI	1	3117 (11566)
SnaBI	1	592 (11566)
XbaI	1	3058 (11566)
XcmI	1	3823 (11566)

Plasmid maps for the vectors used in this study.
